# Evaluating rheumatoid arthritis-associated pulmonary disease in Africa

**DOI:** 10.7196/AJTCCM.2020.v26i4.124

**Published:** 2020-12-01

**Authors:** Richard Raine

**Affiliations:** Respiratory Clinic, Department of Medicine, University of Cape Town, South Africa

**Keywords:** Rheumatoid arthritis, Interstitial lung disease, Ultrasonography

## Editorial

The prevalence of interstitial lung disease (ILD) in rheumatoid arthritis (RA) varies between 7.7 and 67%, depending upon the diagnostic methods available.^[1]^ The paper in this issue by Gbadamassi *et al*. ^[2]^ from Lomé, Togo, suggests that over 70% of patients with rheumatoid arthritis had features suggestive of lung disease. In their group of 28 patients with RA, dyspnoea was present in 47%, with ILD demonstrated on chest radiograph in 18%. Abnormal spirometry was present in 25%. Unfortunately, chest computed tomography (CT) was not available. These figures are not too dissimilar to the findings of Morrison *et al*.,^[3]^ who investigated patients with RA in Cape Town in the early 1990s, prior to the advent of chest CT.

Respiratory symptoms and clinical signs in RA are common but nonspecific. The advent of high-resolution CT (HRCT) has allowed earlier and more precise detection of RA-ILD. The most common patterns identified are usual (UIP) and nonspecific interstitial pneumonitis (NSIP).^[4]^ Traditional diagnostic methods such as lung biopsy can often be avoided if typical HRCT patterns are present. Other diagnostic methods include pulmonary function testing, which is nonspecific for diagnosis but is extremely useful in prognostication.^[5]^

Ultrasound of the lung is now widely used, with ultrasound equipment becoming increasingly available and portable at relatively low cost. Lung ultrasound has been investigated as a screening test for RA-ILD. Moazedi-Fuerst *et al*. ^[6]^ demonstrated pleural nodules or B-lines in 28% of patients with RA studied, HRCT confirmed the presence of early ILD. A further study comparing standard and ‘pocket’ ultrasound devices showed sensitivities of 92% and 89%, with specificities of 56% and 50%, respectively, when compared with HRCT.^[7]^

RA is ubiquitous and many areas of the world do not have ready access to sophisticated radiological techniques such as HRCT. Treatment of RA-ILD requires suspicion and confirmation of diagnosis before introduction of potentially harmful agents such as high-dose corticosteroids, immunosuppressive or antifibrotic therapy. Ultrasound should be investigated further as an accessible tool for use in resource-limited environments.
